# A novel rare event approach to measure the randomness and concentration of road accidents

**DOI:** 10.1371/journal.pone.0201890

**Published:** 2018-08-08

**Authors:** Rafael Prieto Curiel, Humberto González Ramírez, Steven Richard Bishop

**Affiliations:** 1 Department of Mathematics, University College London, London, United Kingdom; 2 LICIT Laboratory, Université de Lyon/ENTPE/IFFSTAR, Lyon, France; Southwest University, CHINA

## Abstract

**Background:**

Road accidents are one of the main causes of death around the world and yet, from a time-space perspective, they are a rare event. To help us prevent accidents, a metric to determine the level of concentration of road accidents in a city could aid us to determine whether most of the accidents are constrained in a small number of places (hence, the environment plays a leading role) or whether accidents are dispersed over a city as a whole (hence, the driver has the biggest influence).

**Methods:**

Here, we apply a new metric, the Rare Event Concentration Coefficient (*RECC*), to measure the concentration of road accidents based on a mixture model applied to the counts of road accidents over a discretised space. A test application of a tessellation of the space and mixture model is shown using two types of road accident data: an urban environment recorded in London between 2005 and 2014 and a motorway environment recorded in Mexico between 2015 and 2016.

**Findings:**

In terms of their concentration, about 5% of the road junctions are the site of 50% of the accidents while around 80% of the road junctions expect close to zero accidents. Accidents which occur in regions with a high accident rate can be considered to have a strong component related to the environment and therefore changes, such as a road intervention or a change in the speed limit, might be introduced and their impact measured by changes to the *RECC* metric. This new procedure helps us identify regions with a high accident rate and determine whether the observed number of road accidents at a road junction has decreased over time and hence track structural changes in the road accident settings.

## Introduction

According to the World Health Organization (with data available at https://bit.ly/2L89d9g), during 2013, more than 1.2 million people died around the world due to a road accident, one of the most frequent causes of death, 2.8 times the mortality due to Malaria and 3.3 times the mortality due to violence. Whilst the number of road accidents is now a global concern, it is, however, possible to either reduce their frequency or their impact: in the UK, for example, the number of road fatalities decreased from an average of more than 3,400 each year between the year 2000 and the year 2004 to an average just above 1,800 fatalities each year between 2010 and 2013 (with data available at https://bit.ly/1JjD4iJ). This dramatic decrease in the number of fatalities in the UK indicates that accidents do not simply just occur and that through sensible policies, thousands of deaths around the world could be avoided.

Broadly speaking, road accidents have three potential causes: firstly, it could have something to do with the *driver*. It was shown that the chances of a driver having an accident are many times higher if he or she consumes high levels of alcohol [1] or is fatigued [2] and accidents are considerably more likely to lead to a fatality if the driver exceeds the speed limit, according to the Royal Society for the Prevention of Accidents (report available at https://bit.ly/2aZioDQ). Secondly, accidents might have something to do with the *local environment*, for example, due to a reduced visibility, the weather conditions, a poorly designed junction, a poorly enforced speed limit, faulty traffic signals and more. Finally, an accident might occur simply due to *(bad) luck*, for example, a non-preventable failure in the car and so on. The first and second causes, attributed to the driver and to the environment, can and should be reduced to a minimum, both in terms of their frequency and their impact.

How do we distinguish whether a certain region has an increased probability of accounting for an accident? Clearly, the road geometry, road obstacles and the level of traffic have an impact on the distribution of road accidents, but these tend to remain unchanged for long periods of time and are specific to a certain area so it makes any comparison between different cities, or even areas of a city, quite complicated.

If, for example, we analyse data and find a specific junction with several accidents, would that be enough to suggest that it is necessary to reduce the speed limit or put in a road intervention scheme? Is there a threshold as to the *acceptable* number of accidents that a street or a road could experience and yet still be considered safe?

### Heat maps and the random location of accidents

Numerous studies have been conducted to identify the spatial patterns of road traffic accidents and develop techniques to identify crash-prone locations using, for instance, Bayesian inference [3], or data mining techniques [4, 5]. A frequently used tool to analyse the location of road accidents (as well as other spatially-distributed events, such as the location of crimes or gang fights) is a heat map [6–10]. This tool provides a graphical description of the location of a point process, which highlights areas or junctions more prone to accidents.

**Fig 1:**
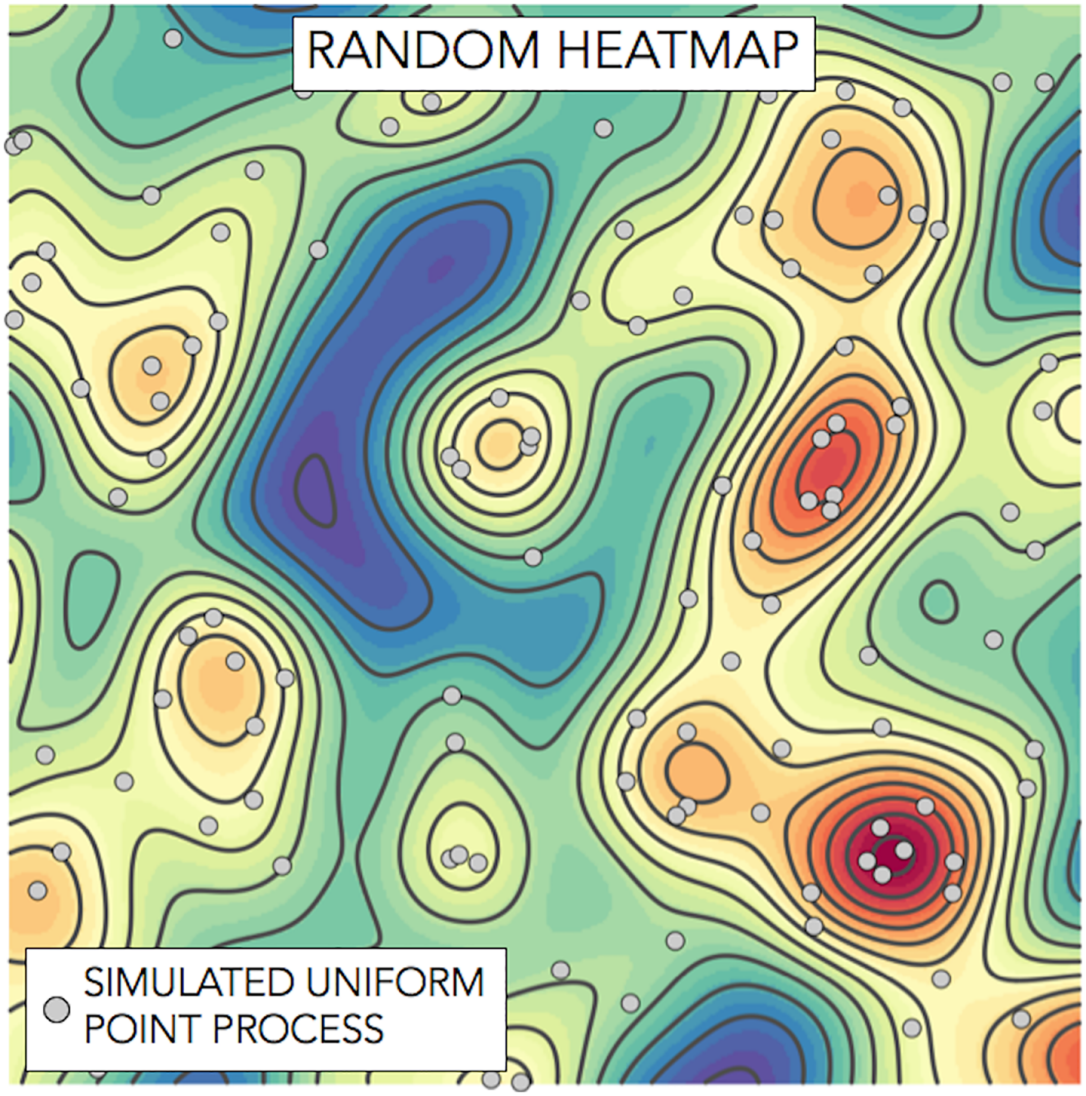
Heat map of a simulated point process that follows a uniform distribution. The underlying uniform distribution has the property that every region is expected to contain a number proportional to its area, thus, any apparent concentration observed in the map and any region with a higher, or fewer, number of points is only the result of randomness and not the result of a higher probability of observing a point in that region.

There are, however, two technical aspects with respect to heat maps which are often ignored: when we say that a location is considered to be “hot”, what are we comparing this with? and to what degree is the observed heat map the result of randomness? The relevance of randomness, in terms of its spatial distribution, is that every point process, no matter how it is generated and whatever the underlying distribution, will result in a set of observations being relatively close to each other, thus, even random points (where the term ‘random’ is used here for a uniform distribution) might be interpreted as having a “hot region” (Fig 1).

Although a heat map offers a visual tool for representing road accidents, it might actually result in misleading conclusions when the random element of the location of road accidents is not considered. The crucial difference between a point process that is generated by a uniform distribution and a point process with a different distribution, is frequently undetectable based on a simple visual inspection. A similar situation occurs when a single road is considered, an apparent concentration of accidents will appear, no matter how random or concentrated road accidents are. A formal statistical test against *Complete Spatial Randomness* can be constructed by considering the distance to the nearest neighbour of each point and compare this against a uniform distribution [11] and only orient efforts at a specific location when spatial randomness is rejected.

### Concentration of road accidents

Road accidents might happen due to a mixture of environmental elements, for example, an obstructed visibility, excessive speed of road users, the curvature or quality of the roads, the street lighting and more. These conditions perhaps repeat, almost under the exact same conditions, day after day and so we expect to observe particular road junctions or segments with a much higher number of accidents than others if the environment is the main cause. However, accidents might also happen because of factors related to the driver or simply because of luck, and the chances are that interventions oriented to the road rather than the driver would not reduce this type of accident. A natural way to detect whether road accidents might be attributed to elements on the road rather than the driver is through its concentration. If there is an element which increases risk related to the environment, then more accidents would occur in this specific location than elsewhere and therefore a high concentration should be observed.

The degree of concentration of events has been shown to play a crucial role in other aspects, such as wealth [12, 13], the population of cities, the size of a forest fire [14] or crime. By considering the victims who suffer crime [15, 16], the offenders who commit them [17–19] and the places in which crime is executed [20, 21], it has also been shown that crime is highly concentrated. In the specific case of the places in which crime is executed, a “Law of Crime Concentration” has recently been developed [20] which provides a relevant reference in the study of crime at places.

Although crime and road accidents are fundamentally different events, they both share a low frequency, a high degree of concentration and the fact that both are, to a certain extent, unpredictable. Thus, both areas of research can utilise the tools developed to deal with their low-frequency but highly-concentrated type of events.

Statistically speaking, one of the things that make road accidents (as well as crime) hard to analyse is their low frequency. In London, for example, the road junction with the highest number of accidents has (just over) one accident every month, which makes them highly unpredictable and statistically hard to deal with. No relevant pattern, in terms of the day of the week or the time of the day of road accidents, can realistically be observed when the frequency of such events is so low. Moreover, since road accidents are low-frequency events, we observe that the majority of road segments (or intersections) suffered no accidents within the time period of the analysis. Hence, the Gini coefficient *G*, which is a popular measure of the degree of concentration [22], based on the count of accidents in each road segment, will reveal a high concentration of accidents, even when they are uniformly distributed amongst the segments in which accidents occurred. In other words, the Gini coefficient obtained directly from the count data does not take into consideration the fact that these events are rare, and will naturally regard the data as having a high degree of concentration. As a consequence, the Gini coefficient of low-frequency events might easily be misinterpreted and might make it difficult to compare the concentration of road accidents between cities or different motorways.

The objective of this work is to present the *Rare Event Concentration Coefficient* (*RECC*), a metric specifically designed for the analysis of low-frequency and highly concentrated data [23] which is comparable between different cities, types of accidents, time periods and times of a day. This tool has been successfully applied in the case of crime concentration [24] and now provides a starting point for the analysis of the concentration of road accidents.

## Spatial counts of the road accidents

Two sources of information and two types of analysis are used here to compare the concentration of road accidents. Firstly, data available from the Transport for London (TFL) website (available at https://bit.ly/295vkak) allows the spatial concentration of road accidents within a city to be measured. Secondly, data available from the Ministry of Transportation from Mexico (available in Spanish at https://bit.ly/2OgAO6C) allows the concentration of road accidents on motorways to be measured. The type of road accident and data from an urban environment is very different from that taken on motorways and therefore, we present two types of analysis based on a different discretisation of the observed road accidents.

Road accident data has, in general, two issues. A considerable number of non-fatal injury accidents are not reported to the police and are therefore not included in the available data, however, issues of under-reported accidents [25] are considered minimal in the case of more severe accidents. Also, there might be a lack precision related to the location of the road accidents, especially in the case of accidents on motorways, as there are fewer reference points. However, no systematic bias on the location of the road accidents should be observed and therefore, concentration metrics give reliable information about the underlying pattern.

### Urban data—London

The data from the Transport for London contains information on road traffic collisions that involve personal injury occurring on public highways which have been reported to the police. Data is collected by the police at the scene of an accident or, in some cases, reported by a member of the public at a police station, then processed and passed on to Transport for London. The data, taken between 2005 and 2014, includes 242,782 unique collisions, with *x*, *y* space coordinates available. Accidents are subdivided into three categories: *Fatal injury*, where death occurs in less than 30 days as a result of the collision, *Serious injury*, if there are fractures or injuries requiring hospital treatment, and *Slight injury*, where the accidents do not require medical treatment. Table 1 contains the reported frequencies between 2005 and 2014.

**Table 1:** Observed frequencies of collisions in Greater London between 2005–2014.

Category	Fatal	Serious	Slight	Total
Frequency	1,670	27,788	213,324	242,782
%	0.7	11.4	87.9	100

For the purpose of taking into account only the most urban parts of the city, only the central area of London is considered here, which accounts for 70% of the road accidents registered by TFL occur.

### Motorway data—Mexico

The motorway data considered here contains road traffic collisions registered on motorways in Mexico. The data is divided for each motorway and considers, for each accident registered by the police, the distance from the starting point of the highway. Unfortunately, the data does not include in which direction of the road the accident occurred.

**Fig 2:**
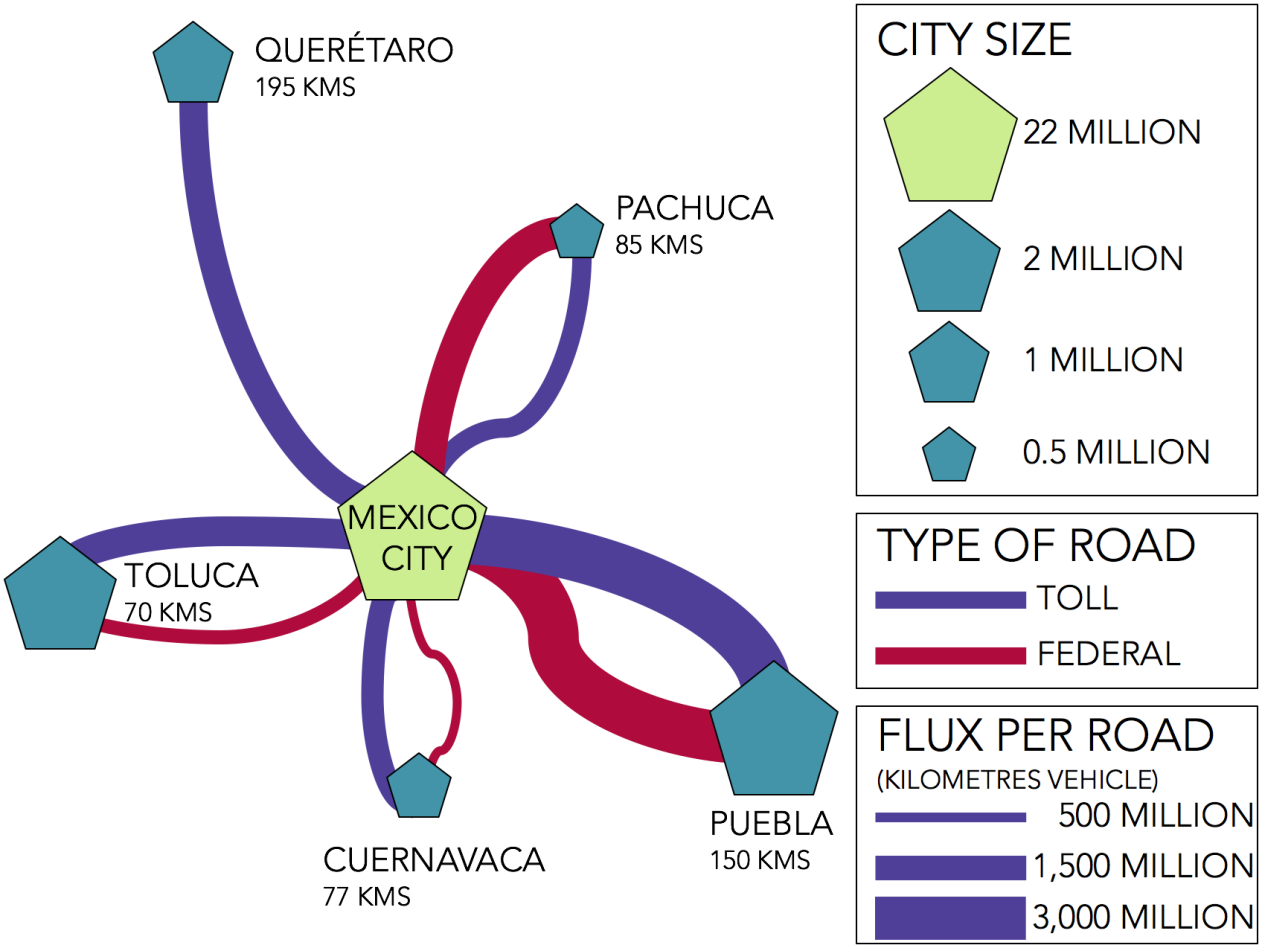
Main roads connecting Mexico City. Schematic representation of the nine roads which connect Mexico City and the five main cities in its peripheral region.

The motorways analysed have Mexico City as their starting point, connecting the capital of Mexico with five large cities: Cuernavaca, Toluca, Pachuca, Puebla and Querétaro (Fig 2). There are two types of motorways, Federal Roads (free of charge) and Toll Roads and each city has both, a Federal Road and a Toll Road connecting them to Mexico City, except for the case of Querétaro for which the Federal Road first passes through another city (Toluca) and so it is not considered. In total, 9 motorways are considered for the study.

The length of the motorway and the vehicle flow rate is different for each of the 9 motorways considered. Both of these factors become relevant when it comes to studying road accidents. Longer roads or those with a higher number of vehicles are expected to have more accidents even if the risk for a driver is the same as compared to a shorter or less used road. Therefore, the flow, measured in *vehicle kilometre per year* units, makes the risk on each road comparable.

Taking into account the length of the road and the number of cars using it, allows a comparison of different roads to be made. For instance, in Table 2 we observe that the Toll Road between Mexico City and Querétaro has the highest number of accidents between 2015 and 2016, yet, is also the longest road among the 9 considered and has a considerably high vehicle flow. The Federal Road between Mexico City and Cuernavaca, on the other hand, has a higher accident risk and is more lethal (meaning that a driver is more likely to suffer an accident and it is more likely that the accident will result in a fatality) than in any other of the roads considered, but it is a short road with a reduced traffic flow and so it does not have as many accidents as the other roads. Thus, comparing the accident risk between different roads has to be based on the length of the road and the number of vehicles that use it or its flow.

**Table 2:** Observed frequencies of collisions on the nine motorways which have Mexico City as origin between 2015 and 2016.

	Federal Road					Toll Road				
Destination	length	flow	accidents	victims	fatal	length	flow	accidents	victims	fatal
Cuernavaca	60.5	467.9	105	159	22	70.7	1204.7	106	128	23
Toluca	55	764.5	117	90	20	55	1589.4	46	45	15
Pachuca	62.5	1761.3	162	161	29	62.5	1083.3	62	99	20
Puebla	121	2736.3	49	63	13	121	2725.7	162	309	46
Querétaro	—	—	—	—	—	164	1548.8	293	362	64

*length* of the road measured in kilometres and *flow in of vehicles* measured in millions of vehicle kilometres per year.

The accident risk (number of accidents per vehicle kilometres of travel) and how lethal the accidents are, varies considerably between different roads. The road with the highest accident risk (the Federal Road between Mexico City and Cuernavaca) is actually 12.5 times more prone to accidents and 9.9 times more likely to have lethal accidents than the safest road (the Federal Road between Mexico City and Puebla).

## Methodology

It is important to determine when two accidents have occurred at the same location. Different levels of data aggregation have been used in previous studies, from countries, provinces, counties, road segments, a point pattern process, road junctions and segments of a road with various lengths [26].

**Fig 3:**
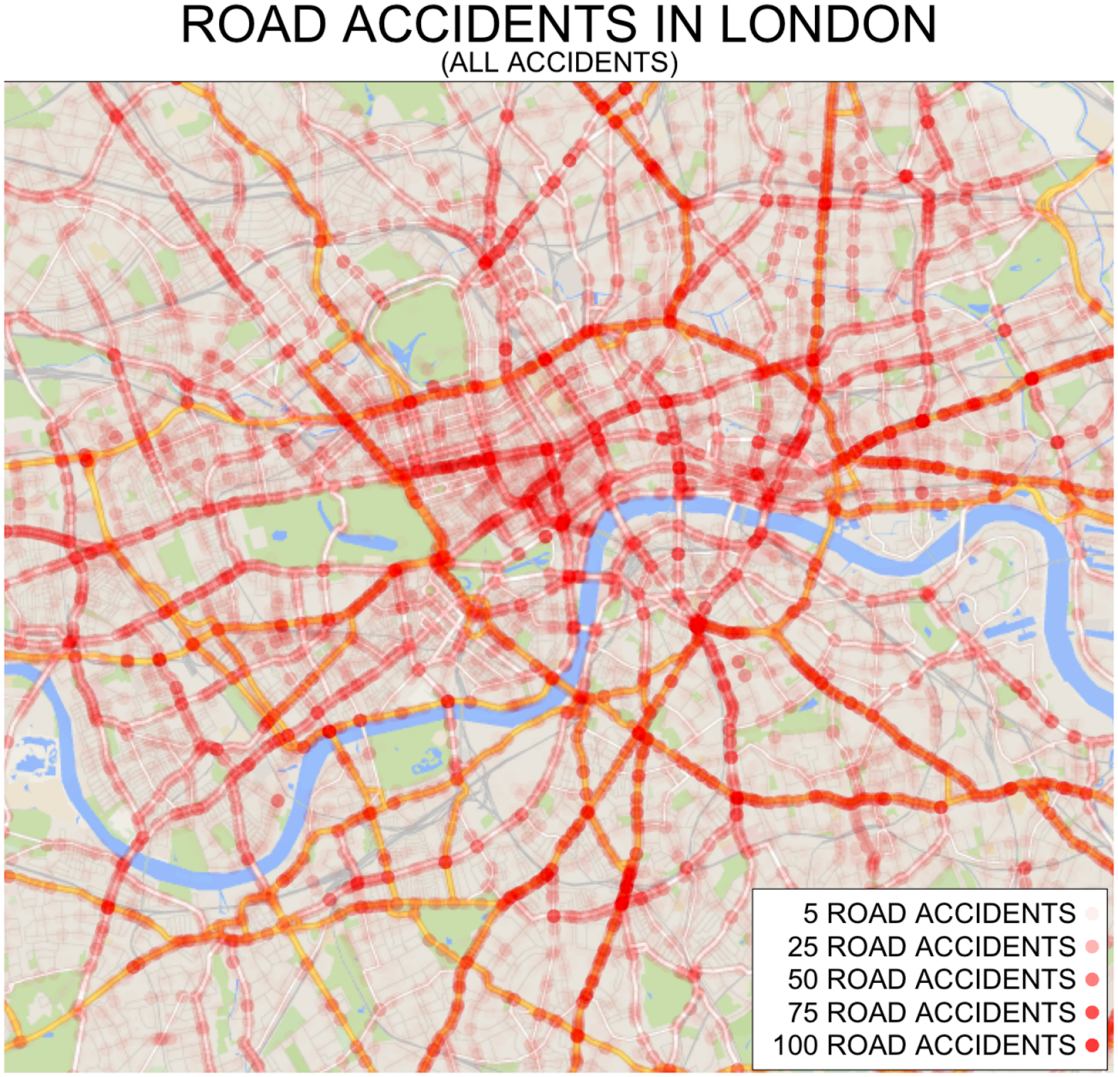
Registered road accidents in Central London between 2005 and 2014.

The hypothesis that road accidents are homogeneously distributed (known as Complete Spatial Randomness or CSR) is easily rejected [27] by measuring the nearest neighbour distance for every road accident [11]. A map of where the accidents occurred during the past ten years, in the case of the London data (Fig 3), shows a very specific pattern, highlighting main roads and congested junctions.

### Discretisation of the data

#### Urban environment

In the case of the urban space, we tessellate the region of analysis, that is, the city is divided into nearly 30,000 non-overlapping, regular hexagons, and the number of accidents within each hexagon is counted. A hexagonal tessellation is frequently used in cartography since it offers advantages in terms of the visualisation [28] and it offers equal-area units and minimal correlation with regularly spaced features, as opposed to a square grid [29]. Hexagons of side length 40 metres provide a useful level of refinement for our analysis dividing the region of central London into *N* = 29,600 tiles. Under this partition, Waterloo Bridge, for example, sits within four hexagons from its extremes on either side of the River Thames. Hexagon tiles are small enough that the region they represent are clearly identifiable and, although they do not match exactly with road junctions, they clearly represent parts of streets. Smaller tiles do not capture the patterns of road accidents and larger tiles tend to blend different regions into the same tile. Also, a similar measure of 40 metres is used for urban data in other studies [9, 30], and so this choice is likely to be close to optimal.

#### Motorway environment

In the case of the motorway data in Mexico, we divide the highway into non-overlapping segments of 500 metres and count the number of accidents within each segment. Due to the precision of the data, smaller segments do not group accidents correctly and larger segments are not refined enough to identify a specific location of a highway. Also, 500 metres has been frequently used in other studies when a highway is partitioned [7, 31], so we use this level of partition for consistency. In addition, although there are some vehicular entrances and exits to the motorways between their origin in Mexico City and their outer destinations, these junctions have a reduced number of vehicles compared to the main roads, we thus consider that through each segment of each motorway, the flow of vehicles is approximately the same.

Although using either a tessellation (in the case of the urban data) or a segmentation of the road (in the motorway data) has its disadvantages (such as a potential autocorrelation of the number of accidents) it does allow a region to be clearly identified, to cluster the accidents that are nearby and to consider different levels of refinement. Using this partition of the space transforms the data into a non-negative discrete variable, rather than a continuous measurement of the location of road accident, which is easier to analyse.

**Fig 4:**
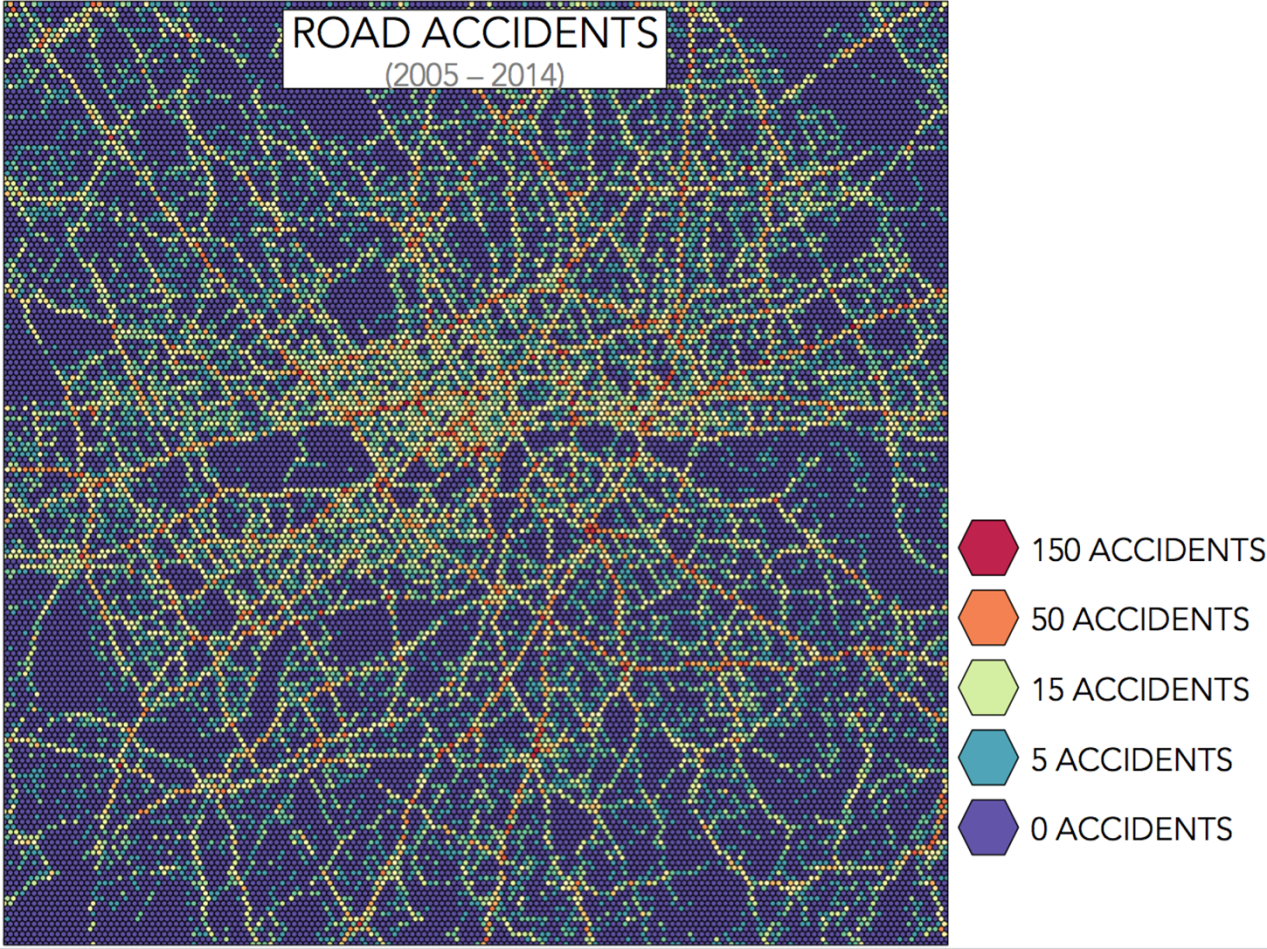
Number of accidents in Central London. Partitioning of Central London into 29,600 hexagonal tiles, with sides of 40 metres, and the count of accidents between 2005 and 2014.

Fig 4 shows the count of the number of road accidents recorded within each tile and the numbers show that there are many tiles with zero, or close to zero, accidents for the ten year period, but there are also a few tiles with more than 150 accidents. The tiling procedure gives comparable observations in terms of the number of accidents that occur, but not in terms of the risk that a driver experience by travelling across each tile since the number of drivers that go across each tile is significantly different. In fact, Fig 4 highlights roads in central London where most casualties occur. If our interest is to explain the reasons why a region has more accidents, a common technique is to divide the number of accidents by the traffic volume, so as to consider the Vehicle Miles of Travel (VMT) [32]. However, our interest here is to determine a measure of the concentration of such events.

### Distribution of road accidents

The number of accidents within each motorway segment or within each hexagonal pixel, during a certain period of time (two years in Mexico and 10 years for the London data), might be equal to zero for obvious reasons (for example, for tiles which overlay a river or a park) or might be much higher in regions with a higher volume of traffic [32]. If suffering an accident in one region does not affect future probabilities of suffering an accident (which might not be true if a road intervention takes place), then the number of accidents suffered in the *i*-th segment or region, *H*_*i*_ say, follows a Poisson distribution with rate λ_*i*_ ≥ 0, where λ_*i*_ is referred to as the *accident rate*, representing the ‘speed’ at which the *i*-th segment or region suffers accidents hence, the number of accidents is simply an observation (or a realisation) from that Poisson distribution [3]. An alternative approach is to use a Negative Binomial distribution [33], by using Survival Theory [34], or other statistical models [25], but here, instead of trying to explain why a region has more accidents (perhaps through a regression technique) we want to measure their spatial degree of concentration, so we simply assume that regions have a different accident rate, without going any further.

Using a Poisson distribution for the number of road accidents observed on each segment has conceptual advantages. Firstly, the expected number of road accidents on a segment is given simply by its rate λ_*i*_. Secondly, it allows us to sum the rates so that the number of road accidents in two segments, *i* and *j*, also follows a Poisson distribution, with rate λ_*i*_ + λ_*j*_. Finally, the number of road accidents over *k* years also follows a Poisson distribution with rate *k*λ_*i*_. Thus, it is easy to interpret the rate λ_*i*_ as the expected number of road accidents in the segment *i*.

In the case of the urban setting, two neighbouring tiles might have similar rates, especially if the same road goes through both of them. In the case of the analysis of motorways, two neighbouring segments might also have similar rates if they experience accidents due to similar causes. Although in our context there is a clear spatial structure that is highly relevant to the problem, we focus on the rates in each of the tiles, and we simply assume that each tile has a fixed accident rate.

With this approach, we move away from the observed count data for road accidents into the analysis of the rates, λ_*i*_, of accidents. What is important is that it is a probabilistic metric, so it considers that a region might have been ‘lucky’ during one year and have only experienced a few accidents, or it might have been ‘unlucky’ and had many accidents. This means that, for instance, if a road segment has no accidents for a year, it does not mean it will never have them in the future, perhaps it is the result of a small rate λ_*i*_. Transforming the observed data of road accidents into probabilities gives a different perspective on its distribution. For instance, consider a road segment *i* with rate λ_*i*_ = 1, so that we expect to observe exactly one accident each year. There is a high probability that the segment will have no accidents for one year, given by exp(−1) = 0.368, and there is also a high probability that the segment will have more than one road accident, given by 0.264, which means that we consider departures from its expected value of 1 accident per year.

#### Inhomogeneous distribution of road accidents

To model the inhomogeneous distribution of accident rates, we assume that the *N* units (either tiles or segments) can be grouped into *k* ≥ 1 distinct groups, where group *j* say, has a relative size of *q*_*j*_ (or, in other words, the group *j* has *Nq*_*j*_ units) and each group has an accident rate λ_*j*_, with *j* = 1, 2, …, *k*. Each one of the *N* units belongs to one and only one group, so that *q*_1_ + *q*_2_ + ⋯ + *q*_*k*_ = 1. To avoid ambiguous definitions, we order the groups by their rate in increasing order, so that λ_1_ < λ_2_ < ⋯ < λ_*k*_. This procedure is known as a mixture model [35] and the (non-parametric) maximum likelihood estimator (*mle*) helps us compute the optimal number of groups in which the units are grouped, denoted by $\hat{k}$ [36], the corresponding accident rate for each group ${\hat{\lambda }}_{j}$ and the relative size of each of the groups, ${\hat{q}}_{j}$. The results of the mixture model (the number of groups, the accident rate and relative size) can be computed using the statistical package CAMAN (Computer Assisted Analysis of Mixtures) by considering the observed number of road accidents suffered in each of the tiles or segments and a test can help us accept or reject the distribution obtained [37].

#### Rare Event Concentration Coefficient *RECC*

The distribution of the rates $\left({\hat{q}}_{1},{\hat{q}}_{2},\ldots ,{\hat{q}}_{k},{\hat{\lambda }}_{1},{\hat{\lambda }}_{2},\ldots ,{\hat{\lambda }}_{k}\right)$ obtained from the data is useful since we could, for example, simulate accidents within each unit to understand the expected departures that simply a natural variability of the number of accidents would yield. In the case of highways, for instance, being aware of the rate of accidents from its origin to its destination gives a full description of the occurred accidents. However, to detect a structural change in the accidents we use the Rare Event Concentration Coefficient (*RECC*) [23]. The *RECC* is defined in terms of the distribution of the rates $\left({\hat{q}}_{1},{\hat{q}}_{2},\ldots ,{\hat{q}}_{k},{\hat{\lambda }}_{1},{\hat{\lambda }}_{2},\ldots ,{\hat{\lambda }}_{k}\right)$ and its expression is given by

$$\begin{array}{c} RECC=\frac{1}{2\sum_{i=1}^{\hat{k}}{\hat{\lambda }}_{i}{\hat{q}}_{i}}\sum_{i=1}^{\hat{k}}\sum_{j=1}^{\hat{k}}{\hat{q}}_{i}{\hat{q}}_{j}\left|{\hat{\lambda }}_{i}-{\hat{\lambda }}_{j}\right|, \end{array}$$ (1)

which is the Gini coefficient [22] of the distribution of the rates. A value of the *RECC* closer to zero is interpreted as road accidents being more homogeneously distributed across the city, and a value closer to one means that road accidents are more concentrated in some regions of the city. The *RECC* is a coefficient comparable over different time periods, between different regions and even for different cities or type of accidents.

The procedure of considering a discrete set of observations, assuming they suffer different rates and then measuring the concentration using the *RECC*, has been used in other contexts, for instance, for the concentration of volcanic eruptions [23] or the concentration of crime suffered by individuals [24].

A procedure to obtain a confidence interval for the observed *RECC* has been developed [24] based on a Monte Carlo method. It assumes that the observed distribution is the *true* distribution and, by simulating road accidents in the road segments, departures from the *RECC* are obtained which could be observed under the same (true) distribution of accidents.

## Results

### Concentration of road accidents in urban environment

**Fig 5:**
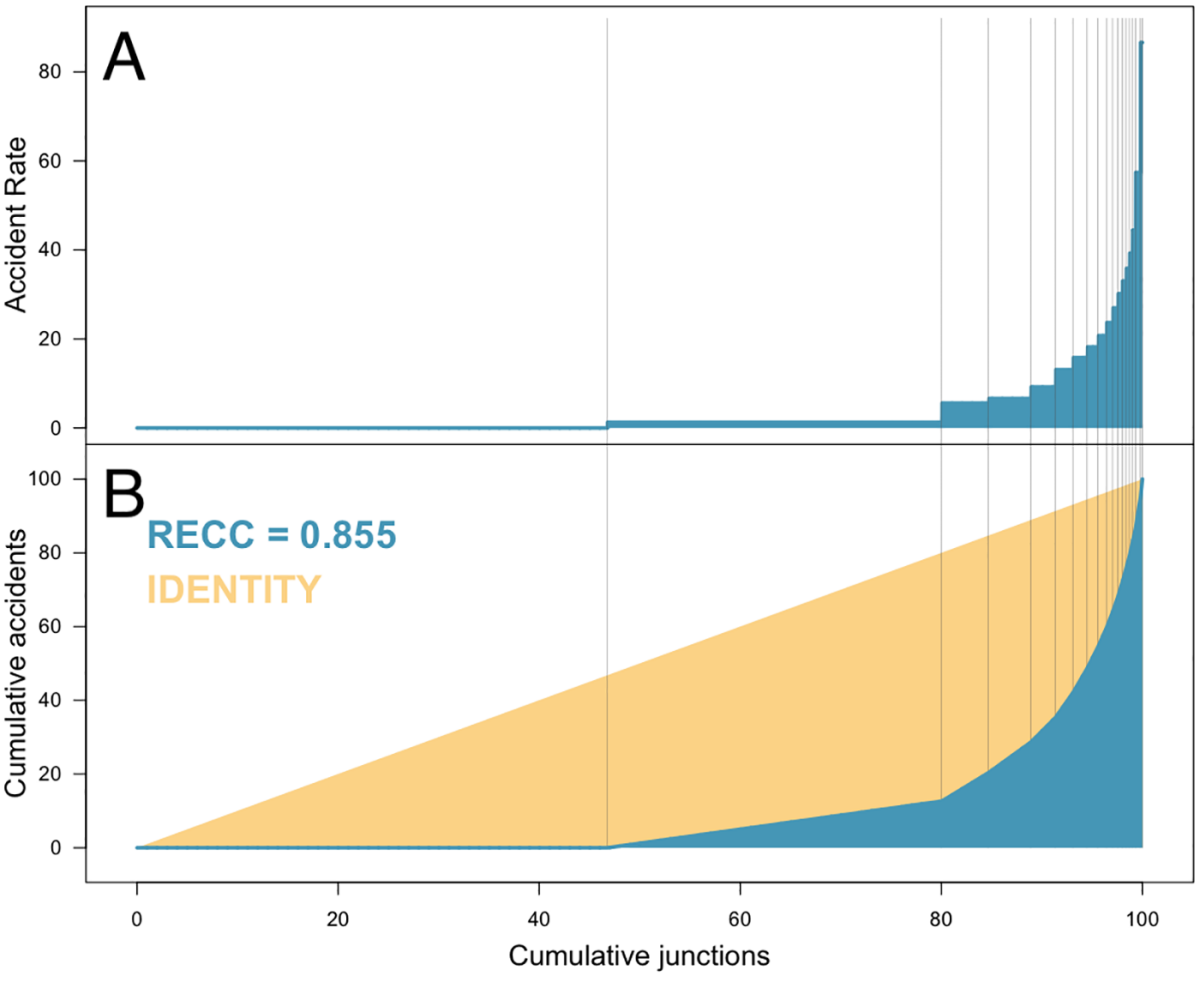
*RECC* of accidents in Central London between 2005 and 2014. A: The accident rate (${\hat{\lambda }}_{j}$) and group sizes (${\hat{q}}_{j}$). B: Cumulative accident rates (blue) and the Lorenz curve (in yellow). The *RECC* is represented by (twice) the area between the two curves.

The Lorenz curve [38] and the *RECC* for the road accidents in London between 2005 and 2014 are displayed in Fig 5 and results indicate that around 47% of the tiles considered have a rate equal to zero (not surprising, since London has lots of parks and a large river passing through it), but also, 33% of the tiles have an estimated rate of ${\hat{\lambda }}_{j}=1.3$, meaning that within the period of ten years, these tiles expect to experience only 1.3 road accidents. These accidents are not considered to be related to the environment, due to the small rate, and so they could have happened anywhere. On the other hand, there are tiles with rates higher than 30 accidents over the ten year period, so they expect to have at least one accident every four months and so on. There is, however, a group of tiles with an estimated rate of ${\hat{\lambda }}_{k}=86.6$, meaning that these tiles expected to have one accident every six weeks.

The level in which road accidents are spatially concentrated is surprisingly high. In Central London, 32% of the accidents happen in only 2.4% of the road junctions, and they get even more concentrated if we focus only on the Serious and Fatal categories. Table 3 shows the *RECC* for the road accidents and results are that fatal and serious accidents tend to be much more concentrated in only a few regions.

**Table 3:** *RECC* metric of the road accidents in London between 2005–2014.

Category	Fatal	Serious	Slight	Total
*RECC*	0.8712	0.8198	0.8057	0.8055

A value of the *RECC* closer to zero means a more homogeneous distribution of road accidents, and a value closer to one means a higher degree of concentration.

Considering only the Serious and Fatal road accidents, results of the mixture model are that around 64% of the tiles have a rate equal to zero. There are, on the other hand, a few tiles (roughly 0.4% of the surface or 109 road junctions) which have an accident rate of almost 11. This means that in the small region represented by the 109 tiles, we expect someone to suffer either a serious or a fatal accident every year. Table 4 shows the distribution of the accident rates.

**Table 4:** Estimated group size ${\hat{q}}_{j}$ and accident rate ${\hat{\lambda }}_{j}$ for the Serious and Fatal accidents in London between 2005 and 2014.

Group	1	2	3	4	5	6	7	8	9	10	11	12
size ${\hat{q}}_{j}$	64.2	22.7	2.1	1.9	1.7	1.5	1.3	1.2	1.0	1.0	1.0	0.4
rate ${\hat{\lambda }}_{j}$	0.000	0.488	0.823	1.159	1.517	1.906	2.337	2.839	3.466	4.359	5.860	10.950

Serious and Fatal road accidents have a surprisingly high degree of concentration. Results of the mixture model are that nearly half of that type of road accident happen in less than 5% of the tiles considered. However, another relevant component of road accidents is that nearly 25% of the Serious and Fatal road accidents occur in tiles in which we expect only one accident every twenty years. Perhaps accidents which occur at road junctions which have such a small rate cannot be attributed to the road itself and the chances are that they occurred due to causes related to the driver (such as alcohol consumption, driving when fatigued or more).

The *RECC* between 2005 and 2014 for the road accidents in London does not show a drastic change in the way accidents are distributed across the city and so a certain stability is observed, despite the decrease in the number of accidents. Results are displayed in Table 5.

**Table 5:** *RECC* for all road accidents between 2005 and 2014 in London.

Year	2005	2006	2007	2008	2009	2010	2011	2012	2013	2014
*RECC*	0.813	0.825	0.824	0.825	0.826	0.831	0.831	0.828	0.828	0.821

Tiles with the highest rates in London have specific environmental factors which contribute to creating more dangerous roads. For instance, certain Underground stations which are transportation hubs, with a large number of pedestrians, are among the tiles with the highest rate in the city: such as Elephant and Castle, Hyde Park Corner and Camden Town. Also, some roads with a high flow have a consistent high accident rate, such as Euston Road and Kingsland Road (the A10 which is a main arterial road) and finally, relevant commercial streets are also among the locations with the highest accident rate, such as Oxford Street.

### Concentration of road accidents on motorways

For the Mexican motorway data, comparing the distribution of the accident rates in the nine highways separately reveals that each road has a different pattern. In the case of the Federal Road between Mexico City and Puebla, the *RECC* = 0.022, meaning that the accidents are distributed almost following a uniform distribution along the whole road. This, however, does not mean that the road expects fewer accidents, but it means that from the origin to the destination, the accident rate remains practically the same at $\hat{\lambda }=0.1978$. One way to interpret this, since the units of observation are segments of a road with 500 metres length and we are using two years of data, is that every 10 years a segment expects to observe one accident. Alternatively, for every 1,263 metres one road accident is expected every year, irrespective of where on the road we start this measure from.

Road accidents are rare events and there is a need to use adequate tools to deal with them. The Federal Road between Mexico City and Puebla has the lowest possible degree of concentration, but it is only when we look at the *RECC* that we are capable of detecting a uniform pattern. A frequently used metric to determine the concentration is the Gini coefficient. Unfortunately, computing the Gini coefficient directly from the number of accidents observed on each road segment is not adequate due to the abundance of observations with zero accidents, since there is a correlation of -0.956 between the Gini coefficient computed in this manner and the average accident rate of the road (the number of accidents divided by the length of the road) meaning that both, the Gini coefficient and the average accident rate give the same information and do not provide any information in terms of the concentration.

Misleading interpretations also might be obtained from the Gini coefficient, directly from the number of road accidents. For instance, in the Federal Road between Mexico City and Puebla gives a value of *G* = 0.8211, in which case, the wrong interpretation would be that accidents in that road are highly concentrated. Furthermore, the Gini coefficient evaluated for the Federal Road between Mexico City and Puebla turns out to be the highest among the nine roads considered here, and hence it can be wrongly concluded that on this road the accidents are more concentrated than on any other road (although looking at the rates, we noticed a uniform pattern).

Accidents have a low frequency and so, in the case of the Federal Road between Mexico City and Puebla we are considering only 49 accidents distributed along 242 units of 500 metres (121 kilometres of road) meaning that, due to the low frequency of road accidents, at least 79.7% of the observations are equal to zero. In general, the low frequency of events (high count of observations with zero events) increases the Gini coefficient: the share of events for a great part of the population is zero, thus meaning more inequality in their distribution. However, by taking into account the distribution of the rates of road accidents in the Federal Road between Mexico City and Puebla and not just the number of road accidents, the results show that almost every segment of that road has the same accident rate and there is practically no concentration of accidents along that road.

Another consequence of the low frequency of accidents is that the Gini coefficient computed directly from the number of accidents tends to give similar results between different roads, with small or negligible differences between them and, in the worst case scenario, with the wrong results and interpretation [23]. To illustrate this, we look at the Gini coefficient of the roads with the lowest *RECC* (the Federal Road between Mexico City and Puebla, with a Gini coefficient of *G* = 0.8211) and the road with the highest *RECC* (the Toll Road between Mexico City and Cuernavaca, with a Gini coefficient of *G* = 0.7401) which shows that using the traditional Gini coefficient, the wrong interpretation that *road accidents have a lower concentration on the Toll Road between Mexico City and Cuernavaca* would be obtained.

Other roads also have a certain degree of uniformity with regards to their accidents. The Federal Road between Mexico City and Pachuca, for instance, has *RECC* = 0.274 and three types of segments are identified: 17% of them have an accident rate equal to ${\hat{\lambda }}_{1}=0$, while 74% of them an accident rate of ${\hat{\lambda }}_{2}=1.36$ and a small segment of the road, 8.2% a rate of ${\hat{\lambda }}_{3}=3.4$, meaning that the majority of the road is to a certain extent risky and only 17% is risk free.

**Fig 6:**
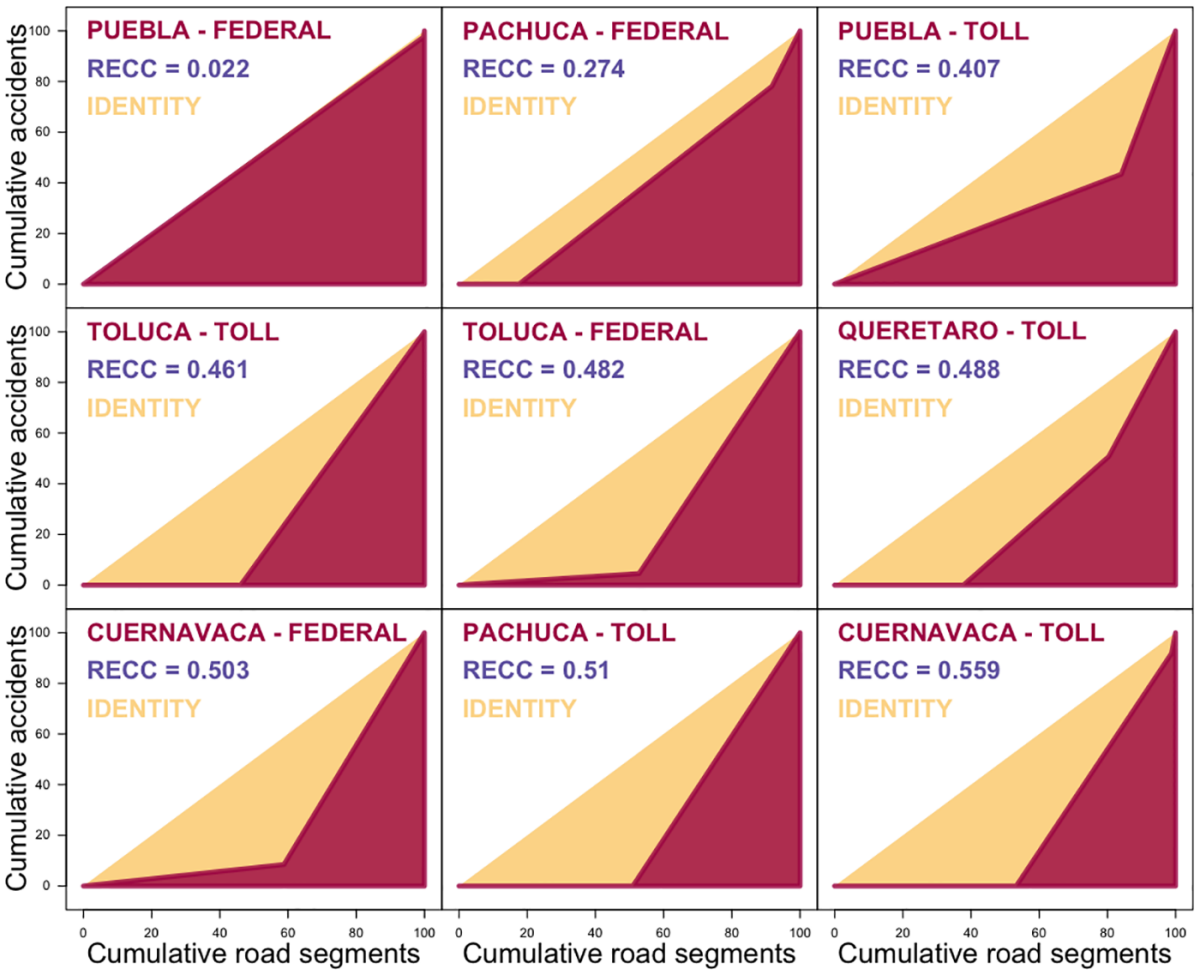
*RECC* of accidents in each of the nine main motorways with origin in Mexico City.

The nine roads in Mexico have a different rate distribution of their accidents (Fig 6). From a *RECC* close to zero, observed in the Federal Road between Mexico City and Puebla, to a *RECC* = 0.559 observed for the Toll Road between Mexico City and Cuernavaca.

**Fig 7:**
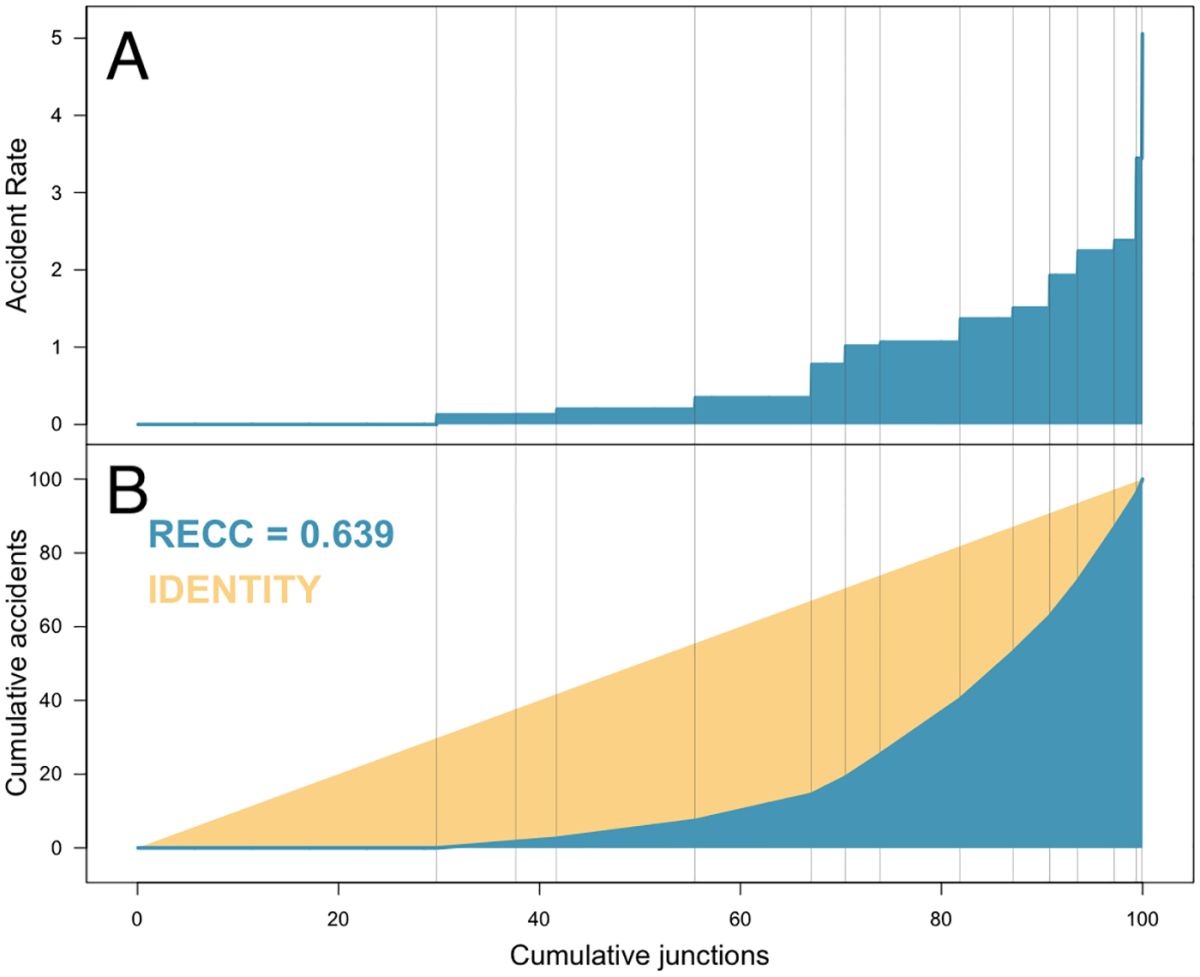
*RECC* of accidents in the nine main motorways with origin in Mexico City considered altogether. A: The accident rates (${\hat{\lambda }}_{j}$) and group sizes (${\hat{q}}_{j}$). B: Cumulative accident rates (blue) and the Lorenz curve (in yellow). The *RECC* is represented by (twice) the area between the two curves.

Computing the *RECC* for the nine roads altogether (Fig 7) shows that nearly 30% of the segments have an accident rate of ${\hat{\lambda }}_{1}=0$ but also, that there are two groups with a high rate. The Federal Road between Mexico City and Pachuca has a set of road segments of five kilometres (not necessarily contiguous) with a rate of ${\hat{\lambda }}_{q}=3.44$, meaning that there is a small number of segments which consists of 5 kilometres of the road in which we expect to observe 17 road accidents each year. Also, on the Toll Road between Mexico City and Cuernavaca, there are two segments (so, one kilometre in length) which have an accident rate of ${\hat{\lambda }}_{r}=5.05$, much higher than in the rest of the nine roads. On that specific kilometre (again, not made of contiguous 500 metres segments) the expected number of accidents each year is more than ten.

Environmental factors that contribute to the chance of having an accident can be identified in the road segments with high accident rates. For instance, among the highest rate segments (${\hat{\lambda }}_{q}=3.44$) of the Federal Road between Mexico City and Pachuca, we find the segments situated at kilometres 35, 72 and 75, where we find the first segment located within an urban area, and the other two segments have junctions. Likewise, we find that the two segments with the highest rate of accidents (${\hat{\lambda }}_{r}=5.05$) of the Toll Road between Mexico City and Cuernavaca, situated at kilometres 56 and 64, are located in a part of the road with a steep slope (7%), with the second segment having a turning place on the high-speed lane (left lane). These local factors: the presence of junctions, the steep slope (7%), and the turning place on the fast lane are likely to be responsible of the high rate of these segments.

The accident rate along these two higher rate sections have been identified using the CAMAN procedure and the *RECC* and this high concentration is attributed to the environment and an intervention to determine whether it is related to the road conditions, its visibility, its design or speed limit should take place.

In total, in these two segments (one on the Federal Road to Pachuca and the other one, on the Toll Road to Cuernavaca) which are less than 0.7% of the 772.3 kilometres of roads considered, there are 4% of the road accidents.

In addition, the different values of the *RECC* observed on the nine roads which originate in Mexico City are not the result of longer roads (so a higher number of observations) or a higher flow, nor as a result of a higher number of accidents, but due to other environmental reasons. For instance, the Toll Road to Toluca and the Federal Road to Puebla both have had less than 50 accidents in two years (in fact, they are the two roads with the lowest number of accidents) but the *RECC* in the first case is 0.461 and in the second case is 0.022, meaning that even when they observe a similar number of road accidents, they follow a different concentration pattern.

## Discussion

A typical approach to determine the concentration/dispersion of a variable (for example, using the Gini coefficient) fails to work as a measure of the concentration of road accidents due to their low frequency and their high level of spatial concentration. The methodology presented here, considering the distribution of the rates and the *RECC*, allows us firstly to overcome the low frequency of events, taking into consideration their random component and to obtain a distribution from which simulations can be easily computed. From the simulations, expected departures from the observed number of accidents can be detected, including outliers.

Results for the urban environment show that road accidents are highly concentrated, especially those that fall into the Serious and Fatal category. This result could be useful to policy makers: by focusing their resources on less than 5% of the road junctions, they are considering the regions where nearly half of that type of accident occurs.

Results for the motorway environment show a much smaller concentration degree. In the case of the Federal Road between Mexico City and Puebla, road accidents are considered to be distributed almost uniformly along the road, meaning that statistically speaking, they have the smallest possible concentration. Also, the procedure introduced here, including the use of the *RECC*, allowed a comparison between different roads and a higher accident rate in two specific segments of the highways was observed (one on the Federal Road to Pachuca and the other on the Toll Road to Cuernavaca). On these specific sections, accidents might be closely related to environmental factors and so perhaps, some of these accidents could have been avoided by a road intervention scheme, such as a reduction in the speed limit.

For a city planner, a quantitative tool such as the *RECC* derived from the mixture model, provides the ability to compare between different severities or over different time periods to determine the effectiveness and impact of a safety program. For events, such as road accidents, which are rare and have a high degree of concentration, a tool which allows valid comparisons between different cities becomes a valuable asset enabling us to learn from past experiences.

The ability to identify regions of a road or of a city which have environmental factors that increase the risk of an accident enables infrastructures to be re-designed accordingly. For instance, in the case of London, these results might be used to justify the plans to transform Oxford Street, one of the London’s roads with the highest accident rate and with the highest number of road fatalities, into a pedestrian street. More information about the transformation of Oxford Street into a pedestrian road is available at https://bit.ly/2h8yOhA. Having identified a segment of a road which puts its users at a higher risk due to its environmental factors, means that something can (and should) be done to reduce that risk.

The methodology presented here could be easily applied to other types of accidents by adjusting the parameters. For example, the tiling procedure could help a risk manager to identify whether there are regions in some industrial complex with an increased rate of an accident, and the *RECC* can be used for purposes other than the analysis of accidents, for example, by monitoring the number of people who required the assistance of the coastguard along different parts of the shoreline or it could be used by an insurance company to determine any changes observed in the distribution of accidents.

## Appendix

### Road accidents in London

The original data is available at the *Transport for London (TFL)* website: https://tfl.gov.uk/corporate/publications-and-reports/road-safety

### Road accidents in Mexico

The original data is available at the *Secretaría de Comunicaciones y Transportes (Ministry of Transportation)* website: http://www.sct.gob.mx/carreteras/direccion-general-de-servicios-tecnicos/estadistica-de-accidentes-de-transito/

### Results

The results of the estimated rates are available on a public repository https://figshare.com/s/3a5201e90cda3930a5ac divided for each motorway in the case of Mexico and for two types of accident severity in the case of London.
